# Anti-Inflammatory Properties of the Citrus Flavonoid Diosmetin: An Updated Review of Experimental Models

**DOI:** 10.3390/molecules29071521

**Published:** 2024-03-28

**Authors:** Yangyang Fang, Wei Xiang, Jinwei Cui, Bining Jiao, Xuesu Su

**Affiliations:** 1 College of Chemistry and Chemical Engineering, Southwest University, Chongqing 400715, China; fangyy2021@email.swu.edu.cn (Y.F.); xiangdv1988@163.com (W.X.); c1701010501@email.swu.edu.cn (J.C.); 2 Key Laboratory of Quality and Safety Control for Citrus Fruits, Ministry of Agriculture and Rural Affairs, Southwest University, Chongqing 400712, China; jiaobining@cric.cn

**Keywords:** diosmetin, inflammation, natural products, NF-κB

## Abstract

Inflammation is an essential contributor to various human diseases. Diosmetin (3′,5,7-trihydroxy-4′-methoxyflavone), a citrus flavonoid, can be used as an anti-inflammatory agent. All the information in this article was collected from various research papers from online scientific databases such as PubMed and Web of Science. These studies have demonstrated that diosmetin can slow down the progression of inflammation by inhibiting the production of inflammatory mediators through modulating related pathways, predominantly the nuclear factor-κB (NF-κB) signaling pathway. In this review, we discuss the anti-inflammatory properties of diosmetin in cellular and animal models of various inflammatory diseases for the first time. We have identified some deficiencies in current research and offer suggestions for further advancement. In conclusion, accumulating evidence so far suggests a very important role for diosmetin in the treatment of various inflammatory disorders and suggests it is a candidate worthy of in-depth investigation.

## 1. Introduction

**Figure 1:**
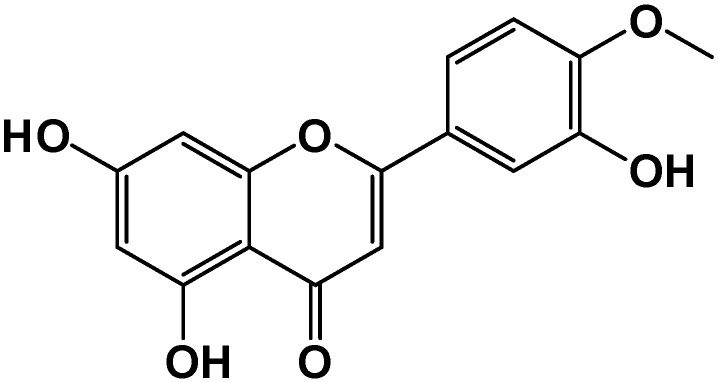
Chemical structure of diosmetin.

Citrus is a well-known fruit worldwide due to its delicious taste, captivating color, and aroma [1]. Flavonoids, essential bioactive components, are abundant in citrus fruits and considered indispensable for various nutritional, pharmaceutical, medicinal, and cosmetic applications [2]. Diosmetin (Dios), 3′,5,7-trihydroxy-4′-methoxyflavone (Figure 1), a bioflavonoid mainly found in sweet oranges and lemons [3], has a broad spectrum of biological activity and attractive properties, such as antibacterial [4], anti-tumor [5], antioxidant [6], anti-inflammatory [7], and estrogenic [8] activities. Among these functions, anti-inflammatory activity is the underpinning principle for many activities since inflammation is the common causative factor for many diseases.

Inflammation is a natural response of the body to infectious agents, which helps to fight off infections and promote the healing of tissue [9]. However, an excessive inflammatory response may aggravate self-injury and can be a contributing factor to chronic diseases including cancer [10], neurodegenerative diseases [11], and cardiovascular diseases [12]. While non-steroidal anti-inflammatory drugs like celecoxib and ibuprofen are commonly prescribed for the treatment of inflammatory diseases, they have dose-dependent side effects that limit their use, especially gastrointestinal injury [13,14]. Therefore, there is an urgent need to develop safer and more cost-effective treatment options. Natural products are generally defined as compounds derived from natural sources, such as plants, animals, and microorganisms that have been used for thousands of years to treat many human diseases [15]. These compounds have historically served as important leads for pharmaceutical companies to develop synthetic drugs. Also, synthetic derivatives of natural compounds with certain enhanced properties can be engineered. In fact, about 34% of U.S. Food and Drug Administration (FDA)-approved medicines are natural products or derivatives of natural products [16]. Groundbreaking discoveries from the first naturally derived medicine morphine, to those of penicillin and streptomycin, and more recent anti-parasitic drugs such as artemisinin [17], show that natural products are definitely the best source of drugs. This review demonstrates the anti-inflammatory properties of Dios to evaluate its potential as a drug for the treatment of inflammatory diseases.

## 2. Anti-Inflammatory Effects

**Figure 2:**
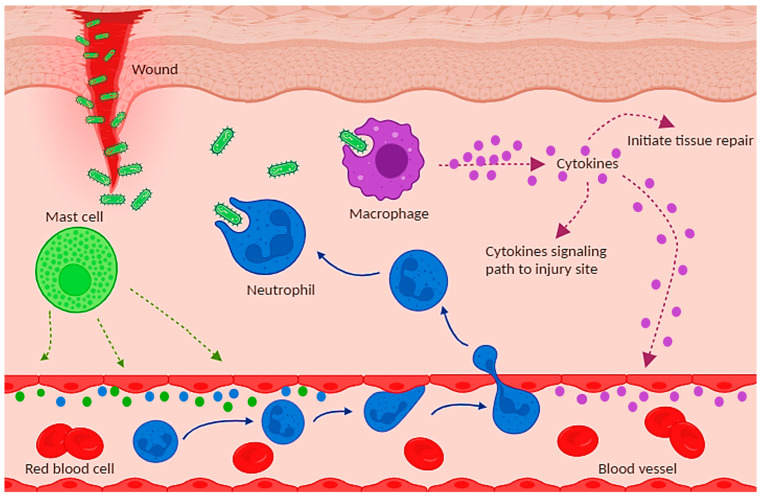
The process of the inflammatory response.

Inflammation is the body’s protective response against infections or injuries, but at the same time it can be a double-edged sword when things go wrong [18]. It can occur in any organ, yet it is most common and also most easily observable in the skin and underlying tissues. An autoimmune disease may result when inflammation targets and destroys the body’s cells. Acute inflammation that fails to stop after the original insult is cleared can become chronic and damaging to healthy tissues [19]. Acute inflammation is initiated when tissue-resident immune cells, such as macrophages, encounter an inflammatory stimulus. This stimulus can be a pathogen, a toxin, or a damaged host cell (Figure 2). Binding of the stimulus to its receptor on the immune cell triggers a signaling cascade that activates the production of cytokines and other inflammatory mediators [20,21]. Inflammatory chemicals dilate blood vessels, increasing blood flow and enhancing vessel permeability, allowing plasma fluid and more immune cells to infiltrate and accumulate in inflamed tissues. This vasodilation leads to clinical signs of inflammation, such as redness, heat, and swelling [22]. The infiltration of blood components into the injured tissue occurs in three phases. The first phase is the exudation of plasma fluid containing various antibacterial mediators, platelets, and blood clotting factors. These factors can destroy microorganisms and prevent any possible bleeding [23]. The second phase is the infiltration of neutrophils—the main phagocytes involved in first-line defense. Once activated by inflammatory mediators, the endothelial cells of blood vessels become adhesive, they attach to neutrophils in the blood flow, slowing them down, before getting them to squeeze through the vessel wall. Chemical cues lead neutrophils to the battlefield, where they engulf bacteria and destroy them with enzymes or toxic peroxides. The pathogen-laden neutrophils then die via apoptosis [24]. In the third phase arrive monocytes. Monocytes differentiate into macrophages, which then remove pathogens, damaged cells, and dying neutrophils by phagocytosis. Macrophages that have completed their task are cleared from the tissue by the lymphatic system [25]. Once the site is cleared from the initial damage, immune cells stop producing pro-inflammatory compounds and, instead, start producing anti-inflammatory mediators, which actively drive the termination of inflammation. This step is essential to ensure a favorable outcome of inflammation. Failure to resolve inflammation leads to the development of chronic inflammation, which continuously causes damage to healthy tissues. Various studies *in vitro* and *in vivo* have been conducted to assess the effectiveness of Dios in attenuating inflammatory responses.

## 3. Cellular Models

### 3.1. LPS-Induced Inflammatory Models

Activated macrophages play an important role in the inflammation response by overproducing pro-inflammatory mediators [26], thus making them a suitable cellular model for evaluating inflammation mechanisms. Lipopolysaccharide (LPS), the outer membrane component of Gram-negative bacteria, is a robust activator of monocytes and macrophages. It is also one of the most effective inducers of the expression of inflammatory mediators used in research, which significantly impacts the levels of inflammatory factors, such as interleukin-1β (IL-1β), IL-6, tumor necrosis factor-α (TNF-α), and IL-8 [27,28]. In a study of human skin fibroblasts treated with LPS, Marcin et al. found that the addition of Dios prior to LPS stimulation was more effective in significantly reducing levels of IL-6 and Il-1β as well as cyclooxygenase-2 (COX-2) and prostaglandin E2 (PGE2) [29]. Additionally, researchers used primary bone marrow-derived macrophages (BMDM) that can be stimulated *in vitro* to proliferate by macrophage colony-stimulating factor (M-CSF) or activated by LPS as a cellular model. They found that Dios, compared to other tested flavonoids, significantly inhibited the proliferation of BMDM in response to M-CSF at low concentrations in a concentration-dependent manner. Meanwhile, Dios inhibited TNF-α and NO secretion in macrophages induced by LPS, especially reducing TNF-α release by approximately 40–55% at 50 μM, which is related to inhibition of the NF-κB pathway [30]. In a separate study, human periodontal ligament cells (HPDLCs) were induced with LPS. The study found that Dios treatment reduced oxidative stress and pro-inflammatory factor secretion by regulating the nuclear factor erythroid-2-related factor 2 (Nrf2)/NF-κB/NOD-like receptor thermal protein domain associated protein 3 (NLRP3) pathway, thereby mitigating periodontitis [31].

### 3.2. Other Cellular Models of Inflammation

Dios has also displayed a potent anti-inflammatory effect in cellular models of inflammation other than LPS-stimulated macrophages (Table 1). For example, in advanced glycation end products (AGEs)-stimulated N-11 murine microglia, Dios has been found to inhibit the production of NO and TNF-α in a dose-dependent manner, which could potentially delay the progression of AGEs-mediated neuroinflammatory diseases [32]. Similarly, in TNF-α-induced human RA fibroblast-like synoviocytes, MH7A cells, Dios treatment decreased the levels of cellular inflammatory factors IL-1β, IL-6, and IL-8. Additionally, Dios treatment inhibited the proliferation of MH7A cells and induced apoptosis, as compared to the control groups [33]. In another cell model of inflammation, rat splenocytes stimulated with quiescent and concanavalin A significantly increased protein levels of COX-2 and inducible nitric oxide synthase (iNOS), and produced inflammatory cytokines. In this study, the expression of both enzymes was significantly reduced following treatment with Dios [34]. However, splenocytes are a mixed cell population and effects on other cell types, especially monocytes, cannot be ruled out [35], thus additional experiments are needed to address this issue. In general, co-culture systems are more conductive than monolayer cell cultures for studying cell–cell interactions in a cellular environment where multiple cells interact with each other [36]. Recently, Lee et al. reported that Dios inhibited pro-inflammatory cytokine production [37] and suppressed adipogenesis-associated protein expression levels (PPARγ, C/EBPα, and C/EBPβ) in a murine macrophage cell line (RAW264.7) co-cultured with a differentiated murine preadipocyte cell line (3T3-L1) [38]. These results could potentially aid in the development of strategies to alleviate and prevent inflammatory disorders associated with obesity. However, evaluating this result in animal models of obesity is necessary.

## 4. Animal Models of Inflammation

Inflammation plays a key role during disease. It is a highly complex but still a very well-coordinated process that is classically triggered by infection or tissue damage. A successful inflammatory response eliminates the trigger followed by a resolution of numerous anti-inflammatory cytokines and lipid mediators for inflammation and tissue repair [39]. However, sustained injurious triggers alter the homeostatic set points, producing several changes in the initial inflammatory process, resulting in collateral tissue damage and organ dysfunction. However, Dios treatment has proven to be a significant aid in organ damage caused by inflammation (Table 2).

### 4.1. Skin

Dios has been reported to have a protective effect on the skin. Atopic dermatitis (AD) is a chronic and recurrent allergic inflammatory skin disease characterized by overexpression of type 2 T helper cells (Th2), cytokines, and serum IgE, and skin itching [58]. Dupilumab, a biological agent, is the Food and Drug Administration-approved IL-4Rα blocker used to treat severe atopic dermatitis in adults [59]. Thus, IL-4 regulation has proven to be a promising target for treating AD. A study conducted on hairless mice using dinitrochlorobenzene (DNCB) found that Dios inhibited IL-4 and LPS signaling pathways and reduced the expression of pro-inflammatory factors like TNF-α, IL-4, and IL-1β in skin lesions. Furthermore, it was confirmed that oral administration of Dios reduced dermatitis scores, ameliorated all symptoms of AD, and decreased the blood index (IgE and IL-4) in serum samples of hairless mice [7,40]. Additionally, increasing the expression of recombinant serine peptidase Kazal type 5 (SPINK5) can improve skin barrier function and improve atopic symptoms [60]. Park et al. found that Dios increased the transcriptional activation of the SPINK5 promoter and regulated events downstream of it to inhibit DNCB-induced skin barrier damage [41].

Psoriasis is another chronic immune inflammatory disease caused by genetic and environmental factors [61]. For its management, effective strategies include inducing apoptosis in keratinocytes and inhibiting the inflammatory response [62]. TNF-α has been proven to cause an increase in cell proliferation of human immortalized epidermal cells (HaCaT) in *in vitro* cell models of psoriasis [63]. In a study, Dios inhibited cell viability and promoted apoptosis of TNF-α-induced HaCaT cells by inactivating the TLR4/NF-κB pathway, which confirmed its anti-proliferative and pro-apoptotic effects in psoriasis. In addition, in an imiquimod (IMQ)-induced psoriasis-like mouse model, Dios ameliorated skin lesions by reducing IL-6 and IL-8 levels, and attenuated inflammatory responses [42].

Understanding the processes that modulate the interactions between the sensory nerves and the skin’s immune system is critical for effective treatments of skin inflammatory diseases [64]. Studies have shown that transient receptor potential vanilloid 1 (TRPV1) is involved in this interaction [65]. The TRPV1 channel contributes significantly to inflammatory skin diseases, especially those induced by UVB radiation exposure [66,67]. Sunburn, a form of cutaneous inflammation caused by UVB radiation, causes changes in both neuronal and non-neuronal systems [65,68]. According to Cho et al., the development of edema in mice ears exposed to UVB radiation is an important sign of skin inflammation [69]. It was shown that Dios, a novel TRPV1 antagonist, mitigated the inflammatory signals induced by UVB radiation [70]. In anesthetized mice, topical treatment with Dios after prolonged exposure of the right ear to UVB radiation effectively inhibited oxidative stress and inflammation in the skin via neuronal and non-neuronal TRPV1 pathways, reduced levels of IL-1β and MIP-2 (a functional analog of IL-8 in humans) cytokines, and reduced ear edema [43]. Thus, Dios is an attractive therapeutic alternative for treating inflammatory skin disorders.

### 4.2. Brain

Inflammation is a common problem in many central nervous system diseases, such as autoimmune diseases, neurodegenerative diseases like Alzheimer’s and Parkinson’s disease, and epilepsy [71]. Bacterial meningitis, on the other hand, has a high mortality rate and can be challenging to diagnose and treat [72]. Even after surviving the initial infection, patients may suffer from long-term neurological disorders [73,74]. Previous research suggested that the best way to treat it is to reduce neuronal apoptosis and inflammation associated with the immune response and bacterial toxins [75]. *Streptococcus pneumoniae* is the most common cause of bacterial meningitis. In a rat model of meningitis induced by *Streptococcus pneumoniae*, Dios treatment was found to decrease concentrations of the pro-inflammatory cytokines TNF-α, IL-1β, and IL-6, and the number of TUNEL-positive cells in hippocampal tissue compared to the negative control group by modulating the PI3K/AKT/NF-κB signaling pathway [56]. Additionally, in a rat model of middle cerebral artery occlusion (MCAO) induced by oxygen-glucose deprivation/reoxygenation (OGD/R), Shi et al. found that Dios alleviated OGD/R-treated PC12 neuronal cell apoptosis, oxidative stress, and inflammation through Keap1-mediated Nrf2/ARE signaling activation and NLRP3 inflammasome inhibition, and attenuated cerebral ischemia-reperfusion (CIR)-induced neurological damage in MCAO rats model [57].

### 4.3. Lung

Lung inflammatory diseases involve complex interactions between structural and immune cells [76]. Acute lung injury (ALI) is a common clinical syndrome that causes diffuse lung inflammation with high mortality rates and has limited therapeutic approaches for its treatment. Studies have mainly focused on oxidative stress and inflammatory responses to understand its pathogenesis and interventions [77]. Liu et al. used the method of intranasal administration of LPS and found that pretreatment with Dios significantly increased the expression of Nrf2 and its target gene HO-1, blocked the activation of NLRP3 inflammation in the lung, and prevented the production of pro-inflammatory cytokines. This effectively alleviated lung histopathological changes [48]. Therefore, Dios may treat ALI via two important mechanisms: scavenging of ROS through Nrf2 activation and the inhibition of inflammation through NLRP3. But further studies are required for a deep insight into these two mechanisms and possible correlation between these pathways. Recently, it has been reported that endothelial cell damage and repair are central to the pathogenesis of ALI [78]. Xia et al. revealed Dios accelerated wound healing and barrier repair by improving the expression of barrier-related proteins, including zonula occludes-l (ZO-1) and occludin, in human umbilical vein endothelial cells (HUVECs) treated with LPS. It is thus inferred that the Rho A/ROCK1/2 signaling pathway played a pivotal role in the acceleration of lung barrier repair. Dios also exhibited a protective effect against lung injury by reducing levels of TNF-α and IL-6 in the serum of mice, thereby reducing alveolar hemorrhage and the accumulation of inflammatory cells [49]. Simultaneously, it is worth noting that peroxisome proliferator-activated receptor-γ (PPAR-γ) is best known for its critical function in controlling exacerbated lung inflammation and injury [79]. And Dios possesses a potent PPAR-γ-activating property [80]. Based on this, Zhou et al. investigated the influence of Dios on inflammation and lung injury triggered by benzo[a]pyrene (B[a]P) stimulation and influenza virus infection. They found Dios activated PPAR-γ, which inhibited the activation of NF-κB and p38 MAPK after exposure to B[a]P, thus alleviating lung histopathological changes and lung injury in mice [50]. These novel findings offered insights into the mechanisms by which B[a]P aggravated influenza virus-mediated lung injury.

### 4.4. Liver

Inflammation in the liver protects this organ from infection and injury, but excessive inflammation may lead to extensive hepatocyte loss, ischemia-reperfusion injury, metabolic alterations, and ultimately, permanent hepatic damage [81]. A central medium of the inflammatory response is the NF-κB signaling pathway, which is also a potential target for hepatoprotective agents. This pathway helps maintain tissue homeostasis, control disease development, and promote cell survival, making it momentous for liver physiology [82]. LPS and D-galactosamine (D-GalN)-induced hepatitis is a well-established model of liver injury promoted by macrophages [83]. In a study, a murine model of endotoxin-induced acute hepatic failure (AHF) was successfully established by intraperitoneal injection of LPS/D-GalN. Yang et al. found that Dios inhibited the expression of phosphorylated IKK, IκBα, and NF-κB p65 in the NF-κB signaling pathway, along with JNK and p38 in the MAPK signaling pathway. Protein levels of the pro-inflammatory cytokines TNF-α, IL-1β, and IL-6, as well as the activities of prostaglandin E2 (PGE2) and COX-2 were also reduced in Dios-treated groups. Furthermore, Dios pretreatment decreased alanine and aspartate aminotransferase activities, thereby easing liver injury caused by LPS/D-GalN, which was significantly different from the untreated group [45].

Non-alcoholic steatohepatitis (NASH) is a chronic liver disease that results from lipid accumulation and inflammation [84,85], and there are currently no FDA-approved drugs to treat it. Lifestyle changes and weight loss are effective approaches, but we need to understand the mechanisms that promote liver injury and NASH inflammation to identify specific therapeutic targets [86,87]. Fortunately, it has been found that Dios can help alleviate NASH [88]. To explore the protective effects and molecular mechanism of Dios against NASH, Luo established models of palmitic acid (PA)-induced HepG2 cells and HFD-induced mice. The results showed that Dios distinctly blocked pathological changes in the livers of HFD-fed mice, reduced TG content and lipogenic protein expression, and suppressed pro-inflammatory factors, such as TNF-α and IL-6. Macrophage chemotactic ligand 10 (CXCL10) and signal transducers and activators of transcription 1 (STAT1) were identified as the central genes by enrichment analysis of liver RNA sequences, which could be the main regulatory targets of Dios. The results confirmed that Dios can regulate lipogenesis and inflammation in a STAT1/CXCL10-dependent manner, but further studies are required to determine how it is inhibited [46]. In addition, environmental contaminants such as nonylphenol (NP) may also promote metabolic diseases and non-alcoholic fatty liver disease [89]. Previous studies have shown that NP administration can lead to oxidative stress through the Keap1-Nrf2 pathway, which can result in inflammation-induced liver damage [90]. Rabia et al. found that Dios treatment successfully reduced levels of inflammatory markers, such as NF-κB, IL-1β, IL-6, TNF-α, and COX-2. Abnormalities in apoptotic markers, endogenous oxidase activities, and underlying histopathological damage in liver tissue were also recovered [47].

### 4.5. Pancreas

Pancreatitis is an inflammatory disease of the pancreas caused by pancreatic duct obstruction, trypsinogen gene mutation, or alcoholism [91]. Unfortunately, there is no specific therapy for acute pancreatitis (AP), which is a severe and often deadly condition [92]. During AP, the NF-κB pathway is activated early in vesicular cells, leading to the expression of multiple pro-inflammatory genes [93]. Among the members of the NF-κB family, p65 is a crucial transcription factor of the classical pathway in AP [94]. In addition, suppression of pro-inflammatory cytokines has been found to ameliorate the severity of AP [95]. Yu conducted a study on the effect of Dios in a well-characterized model of AP induced by cerulean in mice, which closely resembles human AP due to the rapid development of inflammation. The results showed that Dios significantly reduced the production of pro-inflammatory cytokines in serum, inhibited the expression of iNOS proteins in the pancreas, and attenuated pancreatic tissue injury. Furthermore, Western blot analysis showed that Dios treatment significantly attenuated the expression of NF-κB p65 in the pancreatic nucleus during AP, especially at a 6 h time point. Thus, inhibition of NF-κB activation was involved in the mechanism of effect of Dios on AP [44].

### 4.6. Kidney

Inflammation of the kidneys can lead to progressive renal injury, which in turn leads to glomerulonephritis, acute or chronic kidney disease, or end-stage renal disease [96]. Diabetic nephropathy (DN) is a diabetic complication that causes end-stage renal disease [97]. DN occurs due to inflammation and oxidative stress, which can result in increased levels of inflammatory cytokines like TNF-α, IL-1β, and IL-6 in patients with DN [98,99]. Additionally, an animal model of streptozotocin (STZ)-induced diabetic nephropathy showed that elevated Akt levels promote DN by increasing NF-κB [100]. The production of iNOS also facilitates DN by inducing a TLR-2-dependent signaling pathway [101]. A study by Jiang discovered that treatment with Dios attenuated oxidative stress parameters and inflammatory cytokine levels in STZ-induced DN mice. Moreover, Dios treatment significantly reduced the expression of Akt and NF-κB, and reduced iNOS production in the tissue homogenate compared to the negative control group [51]. Therefore, Dios has a protective effect on kidney injury in STZ-induced diabetic nephropathy mice by regulating the Akt/NF-κB/iNOS signaling pathway.

### 4.7. Intestine

Acute and chronic inflammatory diseases of the intestine can cause various health issues and decrease the quality of life of patients [102]. Inflammatory bowel disease (IBD) is a condition characterized by inflammation and oxidative stress, which play critical roles in its pathogenesis [103]. Ulcerative colitis (UC), a form of IBD, can cause debilitating clinical symptoms including diarrhea, rectal bleeding, and abdominal pain [104]. Current treatment strategies for UC include the use of immunosuppressive drugs, anti-inflammatory agents, and biologics [105]. However, their application in clinical practice is limited due to the high rate of relapse and severe side effects, like cramps, abdominal pain, fever, and rashes [106]. A study conducted by Yu investigated the effect of Dios on 2, 4, 6-trinitrobenzene sulfonic acid (TNBS)-induced UC in rats. It was found that Dios treatment led to a dramatic decrease in the secretion of TNF-α, IL-6, and NF-κB, which in turn led to a reduction in colonic mucosal inflammation and colonic ulceration. These findings suggested that Dios has a protective effect against TNBS-induced ulcerative colitis [52]. In addition, Crohn’s disease (CD) is another major form of IBD [107]. It has been demonstrated that elucidating the mechanisms underlying barrier dysfunction and permeability defects has great potential in the treatment of CD [108]. Liu et al. discovered that in LPS-treated colorectal adenocarcinoma (Caco-2) cells and TNBS-induced CD model mice, Dios treatment was effective in decreasing epithelial permeability and improving the expression of proteins related to barrier integrity (such as ZO-1, occludin, and claudin-1) [53]. This indicated that Dios has great potential in the treatment of CD. According to Li et al., Sirt1 can prevent intestinal inflammation by regulating gut microbiota [109]. Circular RNA (circRNA) is known to play a crucial role in the regulation of various diseases, including colitis [110]. Specifically, circ-Sirt1 has been found to inhibit vascular inflammation by regulating NF-κB acetylation and the Sirt1 pathway [111]. Li evaluated the therapeutic efficacy of Dios in treating chronic and acute colitis induced by dextran sulfate sodium (DSS) in mice. Dios was found to significantly ameliorate microscopic colon tissue damage and reduce Sirt1/Sirt1-axis-mediated secretion of pro-inflammatory cytokines IL-1β, IL-6, TNF-α, and COX-2. The protective effect of Dios against intestinal epithelial barrier damage and oxidative stress was also observed in LPS-treated Caco-2 and IEC-6 cells *in vitro* [54]. Therefore, upregulation of circ-Sirt1 to increase Sirt1 signaling may be a potential strategy to counteract DSS-induced colitis.

### 4.8. Reproductive System

Mastitis is a common disease in both animals and humans that refers to inflammation of mammary gland tissue caused by different factors [112,113]. One of the primary causes of mastitis is the invasion of pathogenic bacteria into the mammary gland, with *Staphylococcus aureus* (*S. aureus*) being most common [114]. The activity of myeloperoxidase (MPO) can be used to evaluate the infiltration of neutrophils and determine the degree of inflammation [115]. Studies have shown that Dios treatment can alleviate pathological changes in the mammary gland by reducing MPO levels, pro-inflammatory cytokine release, and NF-κB activation in a dose-dependent manner compared with the *S. aureus* group [55]. In addition, sirtuin 1 (SIRT1) has been reported to have anti-inflammatory effects, and pharmacologic activation of SIRT1 is a promising therapeutic strategy for inflammatory diseases [116,117]. Nrf2 is one of the key downstream target genes of SIRT1 and has been shown to protect against mammary injury during mastitis by blocking ferroptosis [118,119]. Dios was found to upregulate the expression of SIRT1 and Nrf2, providing a new idea for the clinical treatment of *S. aureus*-induced mastitis [55].

## 5. Discussion

**Figure 3:**
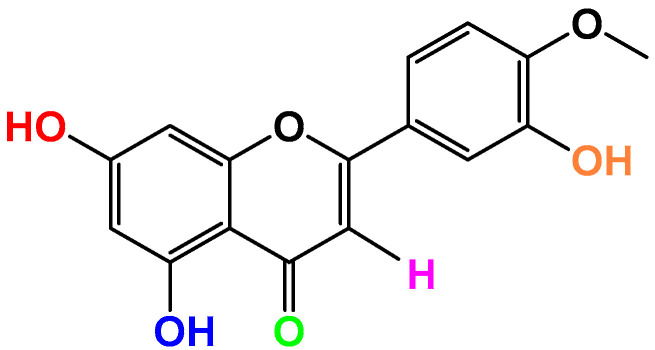
Places of modification of Dios’ structure.

Dios is a citrus flavonoid with a wide range of biological activities, and has demonstrated anti-inflammatory potential under the above-mentioned inflammatory conditions. But the low hydrophobicity of Dios may lead to its poor permeability across intestinal epithelial cells, and reduce gastrointestinal tract absorption, which could decrease its oral bioavailability. The development of highly efficient drug formulations to enhance the solubility of poorly soluble drugs and improve their oral bioavailability is a more promising means of pharmacotherapy than the development of new drug entities. This is an important direction of research because the application of Dios in functional food and medicine is restricted due to its low bioavailability. In the pre-formulation research phase, methods to improve drug dissolution include salt formation, cocrystal formation, or the introduction of polar functional groups into the molecular structure. A series of O-alkyl and O-acyl flavonoid derivatives were efficiently synthesized by T. Kim-Dung Hoang et al. [120]. To evaluate their anti-inflammatory activity, all compounds were tested for their ability to inhibit bovine serum albumin degeneration *in vitro* and carrageenan-induced mouse paw edema *in vivo*. It was observed that acyl derivatives of Dios and hesperetin had more effective anti-inflammatory activity than the control drugs with improved solubility and could be valuable templates for the development of new anti-inflammatory agents. However, research into the structural development of Dios is still scarce, so further research into possible modifications of the structure of diosmetin is recommended. Meanwhile, new derivatives of Dios may obtain more potent anti-inflammatory responses or generate new potential therapeutic targets. Modification positions are marked with colors (Figure 3).

In the formulation design phase, particle size reduction (e.g., solid lipid nanoparticles, nanosuspensions), complexation/solubilization (e.g., use of surfactants and cyclodextrins), and dispersion of the drug in the carrier (e.g., solid dispersions and phospholipid complexes) are viable formulation options to improve the dissolution behavior of poorly water-soluble drugs [121]. Nevertheless, there are currently only a few formulations that apply to Dios. For example, Sun [122] has developed novel lactoferrin-modified long-circulating liposomes for brain-targeted delivery of Dios. Based on the research conducted, it was found that the new form possessed higher bioavailability and a much-prolonged circulation time in rats compared with free Dios. The high brain concentration indicated its excellent effect on brain targeting, having potential implications for Alzheimer’s disease treatment. In addition, a solid self-microemulsifying drug delivery system could improve the solubility and oral bioavailability (4.27-fold) of Dios through transition from a crystalline state to an amorphous state by electrospray technology [123]. To improve the hydrophobicity of Dios, a complex with lecithin was prepared by Brad et al. The conducted research did not answer whether Dios in complex with lecithin is characterized by better bioavailability [124]. Hence, researchers need to combie interdisciplinary knowledge of polymer chemistry, materials science, and pharmacy to design some new drug delivery systems that can flexibly control the dose magnitude and timing, so as to further improve the oral absorption of Dios. More attention should also be paid to systematically evaluate the potential toxicity of these oral formulations and determine the relationship between their efficacy and safety to reasonably guide their effective application.

What’s more, Dios can be tried in combination with more clinical agents to improve efficacy or reduce toxicity. For example, a combination of Dios and 5-fluorouracil (5-FU), a common chemotherapeutic medication used for the treatment of colorectal cancer, was synergized against HCT116 cancer cells, potentially reducing the unfavorable adverse effects of 5-FU. At the same time, it improved anticancer efficacy by inducing apoptosis and blocking mitosis [125]. The results suggest that Dios co-administration may serve as a novel and promising preventive strategy against chemotherapy-induced toxicity.

## 6. Conclusions and Perspective

Inflammation is a major contributor to numerous diseases like cancer, neurodegenerative diseases, and cardiovascular diseases. The flavonoid Dios derived from citrus plants possesses various therapeutic effects, particularly anti-inflammatory properties, making it a potential candidate for drug discovery in several therapeutic areas. By modulating different signaling pathways, particularly the NF-κB pathway, Dios can reduce the secretion of inflammatory mediators. However, the specific mechanism of action is not completely known, and further studies are required to fully understand its molecular targets. The debilitating and devastating effects of inflammation on patients stress the urgent need for safer and more natural therapeutic agents to manage and cure these diseases. Nonetheless, Dios has low bioavailability due to poor solubility and a high first-pass effect [126,127], which limits its development in functional foods and clinical therapeutic products. Strategies to improve its bioavailability include modifying the Dios structure to enhance solubility or permeability, and innovative drug delivery systems to improve intestinal stability. Despite Dios exhibiting promising results, most studies have been conducted in laboratory settings or animal models, and more clinical studies are necessary to determine its effects in humans and recommend safe and effective dosages. As with any dietary supplement or natural compound, it is advisable to consult a healthcare professional before incorporating Dios or any new substance into your health routine.

## Figures and Tables

**Table 1:** A summary of the main anti-inflammatory effects described for Dios in cell culture.

Cell Studies			
Study Model	Dose(s)	Major Findings	Ref(s).
LPS-induced human skin fibroblast cells	150, 300 μM	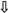 IL-6, IL-1β, COX-2, and PGE2.	[29]
LPS-induced BMDM cells	25, 50, 100 μM	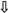 TNF-α, NO, iNOS, and IκB-α phosphorylation.	[30]
LPS-induced HPDL cells	10, 20, 40 μM	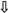 IL-6, IL-1β, TNF-α, and NF-κB/NLRP3 signaling. 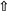 Nrf2 activity.	[31]
AGEs-induced N-11 murine microglial cells	100 μM	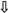 NO and TNF-α.	[32]
TNF-α-induced MH7A cells	5, 10, 20 μM	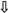 IL-6, IL-8, and IL-1β.	[33]
Quiescent and concanavalin A-induced rat splenocyte cells	50 μM	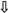 IL-2, TNF-α, IFN-g, COX-2, and iNOS.	[34]
Co-culture 3T3-L1 cells and RAW 264.7 cells	10, 25, 50, 100 μM	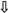 NO, TNF-α, monocyte chemoattractant protein, and iNOS. 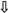 Mitogen-activated protein kinase phosphorylation, and p65 and p50 translocation.	[37]

Symbols: 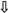 indicates inhibition/reduction, 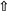 indicates activate/increase.

**Table 2:** A summary of the main anti-inflammatory effects described for Dios in animal models.

Animal Studies				
Study Model	Condition(s)	Studied Sample	Major Findings	Ref(s).
DNCB-induced AD in hairless mice	5 mg kg^−1^ d^−1^ (2 weeks)	Skin	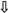 TNF-α, IL-4, IL-1β, iNOS, and MAP kinase phosphorylation (ERK 1/2, p38, and JNK). 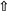 JAK/STAT signaling pathway.	[7,40]
	200 μL 0.5%		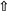 SPINK5 promoter transcriptional activation.	[41]
IMQ-induced psoriasis in mice	5 mg kg^−1^ d^−1^ (1 week)		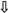 IL-6, IL-8, p65, and IκB-α phosphorylation.	[42]
UVB-induced inflammation in mice	0.01–1% of semisolid formulations		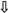 IL-1β and MIP-2.	[43]
Cerulean-induced AP in mice	100 mg kg^−1^	Pancreas	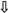 TNF-α, IL-6, IL-1β, iNOS, MPO, TAP, and NF-κB signaling.	[44]
LPS/D-GalN-induced AHF in murine	50 mg kg^−1^ d^−1^ (6 days)	Liver	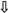 TNF-α, IL-6 and IL-1β. 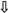 IKK, IκBα, p65 phosphorylation (NF-κB signaling pathway), and JNK and p38 (MAPK signaling pathway).	[45]
HFD-induced NASH in mice	60 mg kg^−1^ d^−1^ (4 weeks)		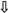 TNF-α, IL-6. 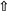 STAT1/CXCL10 signaling via NF-κB.	[46]
NP-induced liver damage in rats	100 mg kg^−1^ d^−1^ (30 days)		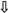 NF-κB, TNF-α, IL-6, IL-1β, COX-2, and anti-apoptotic protein (Bcl-2). 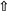 Pro-apoptotic proteins (Bax, caspase-3, and caspase-9).	[47]
LPS-induced ALI in mice	5, 25 mg kg^−1^	Lung	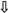 TNF-α, IL-6, IL-1β, and NLRP3 inflammasome. 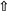 Nrf2/HO-1 pathway	[48]
	5, 10, 20 mg kg^−1^		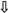 TNF-α, IL-6, and NO. 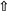 Barrier-related protein expression.	[49]
H1N1 virus and B[a]P-mediated lung injury in mice	50, 100 mg kg^−1^ week^−1^ (27 weeks); 100 mg kg^−1^ d^−1^ (7 days)		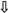 IL-6, IL-8, IP-10, MCP-1, RANTES, TNF-α, COX-2, and PGE2. 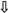 NF-κB and P38 MAPK signaling.	[50]
STZ-induced DN in mice	25, 50, 100 mg kg^−1^ d^−1^ (8 weeks)	Kidney	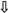 TNF-α, IL-6, NO, Akt, NF-κB, and iNOS.	[51]
TNBS-induced UC in rats	50, 100, 200 mg kg^−1^ d^−1^ (28 days)	Intestine	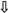 TNF-α, IL-6, and NF-κB.	[52]
TNBS-induced CD in mice	5, 10, 20 mg kg^−1^ (once every other day for 2 weeks)		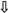 IL-1β, IL-6, and TNF-α. 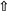 ZO-1, occludin, and claudin-1 expression.	[53]
DSS-induced colitis in a mouse	25, 50 mg kg^−1^ d^−1^ (8 days)		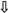 IL-1β, IL-6, TNF-α, COX-2, and acetylated NF-κB via circ-Sirt1/Sirt1. 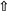 Nrf2 and HO-1.	[54]
*S. aureus*-induced mastitis in a mouse	12.5, 25, 50 mg kg^−1^	Mammary gland	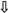 MPO, TNF-α, IL-1β, IκB, and NF-κB p65 phosphorylation.	[55]
*Streptococcus pneumonia*-induced bacterial meningitis in rats	100, 200 mg kg^−1^ d^−1^ (4 days)	Brain	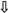 TNF-α, IL-1b, IL-6, Akt, PI3K, MyD88, and NF-κB proteins.	[56]
Cerebral ischemia-reperfusion neurological injury in rats	100 mg kg^−1^ d^−1^ (3 days)		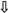 IL-1β, IL-18, and NLRP3. 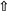 Keap1-mediated Nrf2/ARE signaling.	[57]

Symbols: 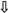 indicates inhibition/reduction, 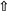 indicates activate/increase.

## Data Availability

The data presented in this study can be obtained from the references.
